# Datasets from fertilized improved and local varieties of cassava grown in the highlands of South Kivu, Democratic Republic of Congo

**DOI:** 10.1016/j.dib.2023.109945

**Published:** 2023-12-13

**Authors:** Wivine Munyahali, Damas Birindwa, Pieter Pypers, Rony Swennen, Bernard Vanlauwe, Roel Merckx

**Affiliations:** a Université Catholique de Bukavu (UCB), DR Congo; b KU Leuven, Dept. of Earth and Environmental Sciences, Belgium; c International Institute of Tropical Agriculture (IITA), Nairobi, Kenya; d International Institute of Tropical Agriculture (IITA), Uganda; e KU Leuven, Dept. of Biosystems, Belgium

**Keywords:** Variety, Mineral fertilizer, Organic input, Cassava growth, Cassava yields, Cost benefit analysis

## Abstract

The use of mineral fertilizer and organic inputs with an improved and local variety of cassava allows (i) to identify nutrient limitations to cassava production, (ii) to investigate the effects of variety and combined application of mineral and organic inputs on cassava growth and yield and (iii) to evaluate the profitability of the improved variety and fertilizer use in cassava production. Data on growth, yield and yield components of an improved and local variety of cassava, economic analysis, soil and weather, collected during two growing cycles of cassava in farmer's fields in the highlands of the Democratic Republic of Congo (DR Congo) are presented. The data complement the recently published paper “Increased cassava growth and yields through improved variety use and fertilizer application in the highlands of South Kivu, Democratic Republic of Congo” (Munyahali *et al*., 2023) [1]. Data on plant height and diameter were collected throughout the growing period of the crop while the data on the storage root, stem, tradable storage root, non-tradable storage root and harvest index were determined at 12 months after planting (MAP). An economic analysis was performed using a simplified financial analysis whereby additional benefits were calculated relative to the respective control treatments; the total costs included the purchasing price of fertilizers and the additional net benefits represented the revenue from the increased storage root yield due to fertilizer application. The value cost ratio (VCR) was calculated as the additional net benefits over the cost of fertilizer purchase.

Specifications Table

**Table:** 

Subject	Agronomy and Crop Science
Specific subject area	Fertilization of cassava varieties using mineral and organic fertilizers.
Type of data	Table
How the data were acquired	Growth parameters, yield and yield component data were collected from trials in farmer's fields conducted during two consecutive years. Data for economic analysis were generated using the purchasing price of inputs (cassava cuttings, mineral fertilizers and farmyard manure) and the market price of fresh storage roots at the time of the experiment. Soil data were obtained through laboratory analysis of soil samples collected before field planting. Soil texture analysis was performed using the LS 13 320 Laser diffraction method, total N and organic carbon using the Dumas combustion method [2]. Available P was measured with the Olsen method [3] while the cobalt-hexamine extraction method [4] was used for cation exchange capacity. Climatic data (rainfall and temperature) were obtained at Lwiro (2.240° S, 28.762° E, 1706 m.a.s.l.), the closest meteorological station to the field trials. Rainfall and temperature data were collected using rain gauges and thermometers, respectively.
Data format	Raw Analyzed
Description of data collection	Field trials in farmer's fields were conducted during two consecutive years (2014 and 2015). A total of 19 trials were planted with 9 trials in the first year and 10 trials in the second year. Two factors were studied, variety and fertilization. The variety factor comprised an improved variety, “Sawasawa” which is resistant to cassava mosaic disease and a local variety, “Nambiyombiyo” which is a high yielding variety commonly grown by farmers in the study area. The fertilization factor comprised mineral fertilizer (N, P, K, Ca, Mg, S and Zn) and organic input (farmyard manure). A field trial was composed of 10 plots (or treatments) of 36 m^2^ each (6m x 6m). Treatments were not replicated within a field, instead each field constituted a replicate per year. Data were collected in a net plot of 16 m^2^ of each plot (4m x 4m). The size of the field trial and number of treatments per trial were the same during the two experimental years. Plant height (distance from the soil surface to the tip of the longest leaf) and stem diameter (at 1 cm above the soil surface) were measured from 5 randomly selected plants at 3-4, 6, 8, 10 and 12 MAP of each growing cycle using a tape and a vernier caliper, respectively. Fresh storage roots and stems were collected from each treatment at 12 MAP, separately weighed using a balance and then extrapolated to a hectare. Data for economic analysis were generated using a simplified financial analysis [5].
Data source location	Data were collected in field trials located in Kalehe at 2.069° - 2.162° S, 28.853° - 28.922° E, 1474 – 1630 m.a.s.l. Kalehe is a territory of the province of South Kivu in the Eastern part of the DR Congo.
Data accessibility	Repository name: IITA Data Bank Yield and yield components data of cassava; https://doi.org/10.25502/bf6e-0181/d Economic analysis data; https://doi.org/10.25502/bf6e-0181/d Soil characteristics data; https://doi.org/10.25502/bf6e-0181/d Rainfall and temperature data; https://doi.org/10.25502/bf6e-0181/d
Related research article	Munyahali, W., Birindwa, D., Pypers, P., Swennen, R., Vanlauwe, B., and R. Merckx (2023). Increased cassava growth and yields through improved variety use and fertilizer application in the highlands of South Kivu, Democratic Republic of Congo. Field Crops Research. 302, 109056. doi.org/10.1016/j.fcr.2023.109056

## 1 Value of the Data

• The database provides a valuable resource describing the growth parameters, yields and yield components of improved and local varieties of cassava
• The database can be used to understand the behavioral responses of cassava varieties to mineral fertilizer and manure application
• The database can be used to compare the performance of cassava varieties in different agroecological conditions
• Nutrient limitations can be identified for site specific fertilizer recommendations for cassava
• The database can be used to understand or evaluate the profitability of the use of improved variety and fertilizer application in cassava production

## 2 Objective

Nutrient limitations due to low soil fertility and insufficient fertilizer use widen cassava yield gaps in the DR Congo. Improved, disease resistant germplasm is indispensable to ensure response to fertilizer [6] while organic matter commonly improves the use efficiency of mineral fertilizer [6,7]. As such, this dataset was generated in order (i) to investigate the effects of variety and combined application of mineral fertilizer and organic inputs on cassava growth and yields, (ii) to identify nutrient limitations to cassava production and (iii) to evaluate the profitability of an improved variety and fertilizer application in a cassava production system in the highlands of South Kivu, DR Congo. The database can also be used for site specific fertilizer recommendations for cassava based on the identified nutrient limitations in the study area.

## 3 Data Description

The application of NPK and farmyard manure (FYM) on improved and local varieties of cassava enables to assess the effects of combined mineral fertilizer and organic inputs on the growth, yield and yield components of both varieties of cassava. It also enables to evaluate the responses of the two cassava varieties to applied fertilizers. Nutrient application consisted of a control without any amendments, the application of full NPK, NPK+(Ca, Mg, S, Zn) and NKP+FYM. To this end, cassava growth parameters including height (H) and stem diameter (D) were measured at 3-4, 6, 8, 10 and 12 months after planting (MAP) while yields and yield components including weights of fresh storage root and stem, number of storage root per plant and weight of storage root piece^−1^ were measured at 12 MAP. These data were collected in 19 trials carried out in two experimental years (9 trials in the first year and 10 in the second year) in the highland conditions of South Kivu and are presented in the files entitled “Variety & fertilizer_Effect _Data and Nutrient response_Data”.

To evaluate the profitability of the use of improved variety and fertilizers (mineral and organic fertilizers), a simplified financial analysis was performed using the purchasing price of inputs (mineral fertilizers and farmyard manure) and the farmgate price of the fresh storage roots of cassava at the time of the experiment. The value cost ratio (VCR) calculated as the additional net benefits over the cost of fertilizer purchase was considered favorable when exceeding 1.18 USD USD^−1^ [5]. The additional net benefits represented the revenue from the increased storage root yields due to fertilizer application. These data were generated from storage root yield data collected in 19 trials (see above details) and are presented in the files entitled “VCR_Variety and VCR_Nutrient response”.

Soil data were obtained through laboratory analysis of soil samples collected from 18 field trials among the 19 trials conducted in the two experimental years (9 trials in each year). These soil samples were taken before establishment of the trial in each field. The physico-chemical properties analyzed include soil pH, total nitrogen, organic carbon, available phosphorus, exchangeable potassium, calcium, magnesium, sodium and manganese, cation exchange capacity and clay, sand and silt content and are presented in the file intitled “Data_Soil characteristic_Field experiment”.

Daily rainfall and temperature data of the two experimental years were obtained at the meteorological station of Lwiro and are presented in the file intitled “Rainfall_Temperature_Data”.

## 4 Experimental Design, Materials and Methods

### 4.1 Experimental design

Twenty-one trials were planted in farmer fields in 2014 and 2015, with 11 and 10 trials in the first and second year, respectively. However, two fields were lost (one in each year) due to animal damage. The experiment was set up as a multilocational trial. Treatments were randomly located in a field and not replicated within each field; instead, farmer fields per site and year were considered as replicates.

Plots measured 6 m x 6 m with a net harvest area of 4 m x 4 m.

### 4.2 Materials and methods

“Sawasawa” and “Nambiyombiyo” are the two cassava varieties evaluated. The former variety is an improved variety-resistant to cassava mosaic disease and was introduced in DR Congo in 2003 by IITA [8] while the latter is a local variety commonly grown by farmers in the study area.

Fresh cuttings of 25 cm were planted at 1 m by 1 m making a total of 10,000 plants per ha^−1^.

Land preparation and weed management were manually done using a hand hoe.

Treatments included a control, NP, NK, PK, NPK, NPK+(Ca, Mg, S, Zn), farmyard manure (FYM), and NPK+FYM with the improved variety and a control and NPK+FYM treatments with the local variety.

Fertilizer rates were 100-22-83 kg ha^−1^ [9,10]. N was split-applied as urea, half at planting and half at 3 months after planting (MAP) while P was applied in the planting hole as triple superphosphate (TSP) and K was split-applied as potassium chloride, half at 1 MAP, and half at 3 MAP. Ca was applied as CaCO_3_ at a rate of 100 kg Ca ha^−1^ while Mg was applied as MgSO_4_ at a rate of 40 kg Mg ha^−1^. S was applied as MgSO_4_ and ZnSO_4_ at a rate of 10 kg S ha^−1^ while Zn was applied as ZnSO_4_ at a rate of 5 kg Zn ha^−1^. FYM was mixed with the soil before planting and applied at a rate of 10 t ha^−1^.

Germination data were collected at 1 MAP while plant height and stem diameter were measured at 3-4, 6, 8, 10 and 12 MAP. Cassava was harvested at 12 MAP. Stem and storage roots were separately weighted to determine cassava yields. Thereafter, storage roots were divided into large marketable and small non-marketable storage roots, counted and weighed. Fresh harvest index (HI) was calculated based on the total weight of stem and storage root at harvest [Fresh storage root weight / (Fresh storage root weight + Fresh stem weight) x 100].

### 4.3 Soil characteristics data

Prior to the installation of a trial, a composite soil sample from at least 10 random sampling spots per field was collected with an Edelman auger at 0-30 cm topsoil, air-dried, passed through a 2 mm sieve and analyzed for standard physico-chemical properties. All analyses were conducted in the laboratories of the Division of Soil and Water Management at KU Leuven, Belgium.

### 4.4 Economic analysis

Value cost ratio (VCR) was calculated as the additional net benefits over the cost of fertilizer purchase [5]. Additional benefits were calculated relative to the respective control treatments. The total costs included the purchasing prices of fertilizer (in 2014 and 2015) obtained from a local agro-dealer in Bukavu (1.3 USD kg^−1^ of urea, 1.4 USD kg^−1^ of TSP and KCl, 2 USD kg^−1^ of MgSO_4_, 6 USD kg^−1^ of ZnSO_4_ and 0.2 USD kg^−1^ of CaCO_3_) and from a farmer in Kalehe (2.5 USD for 100 kg of FYM). The additional net benefits included the revenue from the increased storage root yields due to fertilizer application. The price of fresh storage roots was obtained from local markets in Kalehe, and equaled to 0.40 USD kg^-1^ in both years.

### 4.5 Rainfall and temperature data

Rainfall and temperature data were collected using rain gauges and thermometers, respectively.

### 4.6 Statistical analysis

Analysis of variance was conducted to determine the effects of (and interaction between) the cassava variety and fertilizer application in the two years in Kalehe using a mixed linear model (MIXED procedure, [11]). The effects of the different factors were compared by computing least square means and standard errors of difference (SED); significance of difference was evaluated at *p*≤0.1, *p*≤0.05 and *p*≤0.01. Cassava variety and fertilizer application were considered as “fixed factors” while farmer's field within site and year were considered as random factors in the mixed model analysis. Linear regression was done using the REG procedure [11].

## Ethics Statements

This work included plant material and soil, and did not include work involved with human subjects, animal experiments or data collected from social media platforms.

## CRediT authorship contribution statement

**Wivine Munyahali:** Conceptualization, Methodology, Software, Formal analysis, Investigation, Data curation, Writing – original draft, Visualization. **Damas Birindwa:** Formal analysis, Software, Writing – review & editing. **Pieter Pypers:** Formal analysis, Software, Writing – review & editing, Visualization, Supervision, Validation. **Rony Swennen:** Conceptualization, Methodology, Writing – review & editing, Supervision, Validation. **Bernard Vanlauwe:** Writing – review & editing, Supervision, Validation. **Roel Merckx:** Conceptualization, Methodology, Writing – review & editing, Funding acquisition, Project administration, Supervision, Visualization, Resources, Investigation, Validation.

## Data Availability

Datasets on yield components of fertilized improved ad local varieties of cassava grown in the highlands of South Kivu, DR Congo (Original data) (IITA Data Bank)
